# Boron isotope fractionation in magma via crustal carbonate dissolution

**DOI:** 10.1038/srep30774

**Published:** 2016-08-04

**Authors:** Frances M. Deegan, Valentin R. Troll, Martin J. Whitehouse, Ester M. Jolis, Carmela Freda

**Affiliations:** 1 Department of Earth Sciences, Centre for Experimental Mineralogy, Petrology, and Geochemistry (CEMPEG), Uppsala University, SE-752 36, Uppsala, Sweden; 2 Department of Geosciences, Swedish Museum of Natural History, SE-104 05, Stockholm, Sweden; 3 Istituto Nazionale di Geofisica e Vulcanologia (INGV), 00143 Rome, Italy

## Abstract

Carbon dioxide released by arc volcanoes is widely considered to originate from the mantle and from subducted sediments. Fluids released from upper arc carbonates, however, have recently been proposed to help modulate arc CO_2_ fluxes. Here we use boron as a tracer, which substitutes for carbon in limestone, to further investigate crustal carbonate degassing in volcanic arcs. We performed laboratory experiments replicating limestone assimilation into magma at crustal pressure-temperature conditions and analysed boron isotope ratios in the resulting experimental glasses. Limestone dissolution and assimilation generates CaO-enriched glass near the reaction site and a CO_2_-dominated vapour phase. The CaO-rich glasses have extremely low δ^11^B values down to −41.5‰, reflecting preferential partitioning of ^10^B into the assimilating melt. Loss of ^11^B from the reaction site occurs via the CO_2_ vapour phase generated during carbonate dissolution, which transports ^11^B away from the reaction site as a boron-rich fluid phase. Our results demonstrate the efficacy of boron isotope fractionation during crustal carbonate assimilation and suggest that low δ^11^B melt values in arc magmas could flag shallow-level additions to the subduction cycle.

## Carbon and boron cycles

Carbon is transferred from the Earth’s interior to the surface mainly by CO_2_ degassing at arc volcanoes^1^,^2^. Arc CO_2_ emissions represent a mixture from several volatile sources including mantle carbon, subducted carbon from altered ocean crust or oceanic sediments, and possibly also carbon released from the over-riding plate during final magma ascent^1^,^2^,^3^,^4^. Carbonate is the main CO_2_-carrying mineral phase and its stability at sub-arc depths is debated. Some authors argue that carbonate is unlikely to efficiently break-down under normal subduction conditions^5^, while others propose that carbonate can be efficiently recycled via *e.g.*, interaction with aqueous fluids^6^,^7^. Carbon dioxide outgassing has varied considerably throughout Earth history‒during the warmer Cretaceous period, for example, it has been calculated that there was as much as 220 to 550% CO_2_ outgassing relative to present day rates^8^,^9^. If this calculation is correct, then two important implications arise; (i) that intensified global continental arc activity may have led to excess degassing at paleo-arcs to explain paleo-climate fluctuations and (ii) that decarbonation reactions in the Earth’s crust may be an important, albeit time-variable, factor in modulating the carbon cycle (*cf.* refs 3, 4, 8, 9, 10). It is the latter aspect in particular that provides the incentive for this study, wherein we investigate how crustal decarbonation can potentially affect volatile systematics, and especially boron cycling, in subduction zones.

## Boron as a tracer in subduction zones

**Figure 1:**
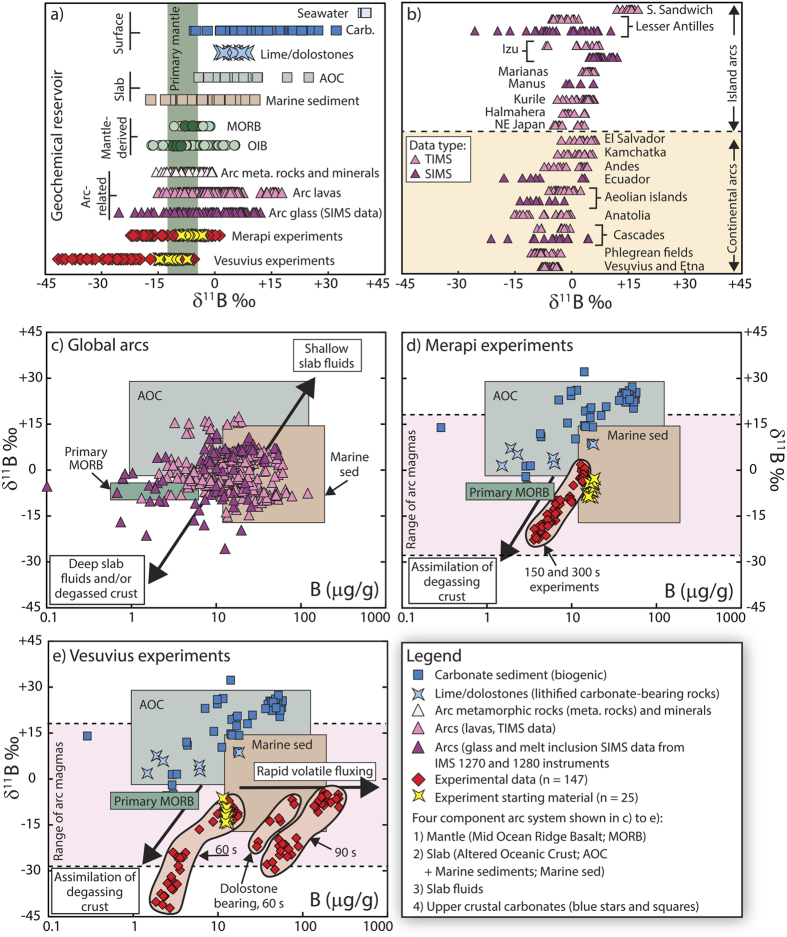
Overview of existing boron data and new data from this study. (**a**) Literature data for various geochemical reservoirs compared to our experiments. The full range of Mid-Ocean Ridge Basalt (MORB) and Ocean Island Basalt (OIB) literature data is shown and the δ^11^B values suggested to represent the primary mantle are highlighted (after refs 41 and 42). (**b**) Literature δ^11^B data for arc volcanoes. (**c–e**) A four component arc system comprising mantle, slab lithologies, slab fluids, and upper crustal carbonates is shown. Process arrows indicate the effects that shallow slab fluids versus assimilation of degassing crust would have on δ^11^B values and B concentration of erupted products. The plotted experimental data correspond to limestone-bearing experiments except for panel e) which shows dolostone experimental data. Error bars (1σ) are smaller than symbol size. Full data sources are provided in Supplementary Table S-5. Abbreviations: TIMS, thermal ionisation mass spectrometry; SIMS, secondary ionisation mass spectrometry.

Boron is a fluid-mobile trace element and its isotope ratios have been used to evaluate the involvement of dehydrated slab materials in arc magma-genesis for the past ca. 25 years (see ref. 11 for a review of current concepts in boron isotope systematics in subduction zones). Boron isotopes are well suited to this task because ^11^B is preferentially fractionated into co-existing fluids, giving rise to boron-enriched slab-derived fluids with high δ^11^B values that progressively evolve towards low δ^11^B values due to continual dehydration reactions and boron loss along the down-going slab^11^,^12^,^13^,^14^,^15^,^16^. This subduction-driven isotopic evolution is mirrored in the boron isotope composition of exhumed rocks and minerals subjected to prograde blueschist facies metamorphism, which generally have negative δ^11^B values due to loss of “heavy” boron into a C-O-H fluid phase (Fig. 1a; see also ref. 16). Thus, it is envisaged that the tendency for many arc magmas to display boron concentrations higher than typical Mid-Ocean Ridge Basalt (MORB) mantle is due to input of slab-derived boron and that isotopically diverse fluids produced during subduction can react with the mantle wedge to generate the wide range of δ^11^B values recorded in global arc suites (*e.g.*, ref. 11 and Fig. 1a–c). A controlling factor on the boron isotope systematics of arcs is likely related to the thermal structure of a particular subduction zone whereby boron-enriched, high δ^11^B fluids will be generated at shallow depths along “hot” slabs and hence low δ^11^B components are likely to be subducted to greater depth (*e.g.*, ref. 17). In contrast, in “cooler” regimes, high δ^11^B components may be preserved and transported to deeper parts of a subduction zone^11^.

Although great progress has been made in understanding the subduction-scale systematics of boron, the processes that may serve to alter the boron isotope composition of magma after segregation from its source, such as crustal assimilation in the over-riding plate, have been addressed less frequently than source-related processes (some notable examples include refs 18, 19, 20). Because BO_3_^3−^ (borate) is thought to substitute for the CO_3_^2−^ site in carbonate^21^, and as carbonate has the potential to strongly degas in magma^22^,^23^,^24^,^25^, we aim to test if boron transport could be tied to carbonate degassing in active subduction zones where the upper plate contains CaCO_3_-bearing minerals. To do this, we offer high temperature-high pressure experimental simulations of carbonate assimilation at conditions corresponding to the over-riding plate and provide spatially controlled boron isotope analyses of the experimental products by Secondary Ionisation Mass Spectrometry (SIMS).

## Results and Discussion

Our experiments were designed to simulate CO_2_ fluxing in magma at mid- to upper-crustal pressure conditions whereby solid carbonate (mainly CaCO_3_ but also MgCa(CO_3_)_2_ in some cases) was allowed to react with pre-fused, powdered magmatic rock from Mt. Merapi (Indonesia) and Mt. Vesuvius (Italy) at 1200 °C and 0.5 GPa for up to 300 s (refs 24 and 25). These volcanic systems were chosen because both are subduction-related and display evidence for crustal carbonate assimilation in form of erupted calc-silicate xenoliths and chemical signatures in erupted rocks and fumarole gas^22^,^23^,^26^,^27^. The advantage of our experiments is that they simulate short-term disequilibrium reactions in order to capture the temporal evolution of magma-carbonate interaction as time variable “snapshots”. Major element compositions of starting materials and experimental products are provided in Supplementary Tables S1 and S2. Boron data for the fused starting materials (*n* = 25) and the experimental products (*n* = 147) are provided in Supplementary Tables S3 and S4.

**Figure 2:**
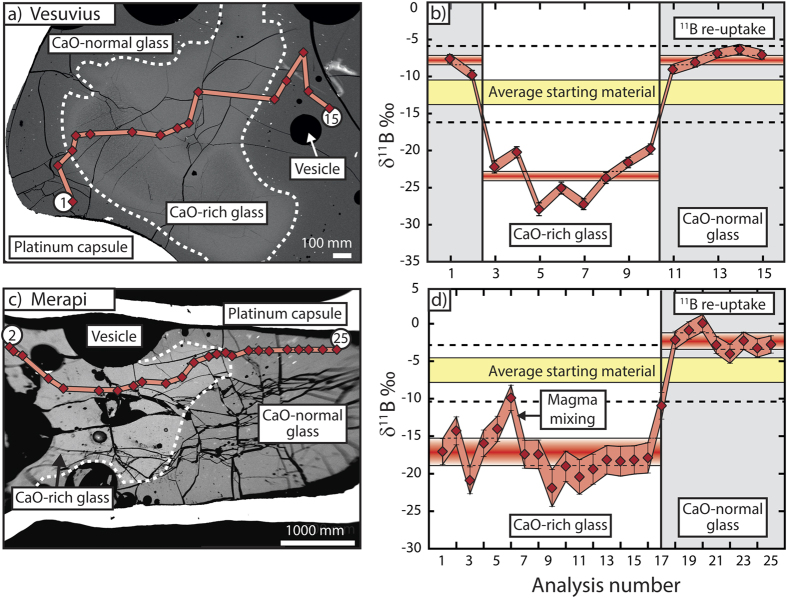
Experimental data. Back scattered electron (BSE) images and δ^11^B profiles for representative (**a,b**) Vesuvius and (**c,d**) Merapi experiments. The solid red line on the BSE images (**a,c**) represents the SIMS traverse and the red symbols indicate analysis spots. In (**b**) and (**d**), average δ^11^B values for different glass compositional domains are represented by red horizontal bars and the full range of starting material values measured is indicated by black dashed horizontal lines. The CaO-rich glasses have significantly lower δ^11^B values than both the starting material and the CaO-normal glass due to transport of ^11^B away from the reaction site in the CO_2_ vapour. Error as in Fig. 1.

Our experimental products comprise a CaO-normal glass similar in composition to the starting materials, a CaO-rich or MgO-rich glass, and a mixing interface between the two domains that shows variable CaO and MgO contents^24^,^25^. Incongruent break-down of carbonate produced free CO_2_ bubbles that permeated all melt domains^28^ (Fig. 2). The δ^11^B values of the starting materials range from −8.8 to −3.5‰ for Merapi and from −14.6 to −7.6‰ for Vesuvius (Fig. 1a). In the Vesuvius case, the measured δ^11^B values overlap with the lower end of the established Vesuvius range (−7.6 to −6.3‰; ref. 29), and are similar to literature δ^11^B values for other Italian magmatic systems (*e.g.*, −13.7‰ at Stromboli; ref. 30). The boron concentration of the Vesuvius starting glass ranges from 12 to 14 μg/g, and is hence close to the reported range of 14 to 36 μg/g for Vesuvius erupted products^29^. To the best of our knowledge, there are currently no published δ^11^B data for Merapi. Boron concentration of our Merapi starting glass ranges from 15 to 18 μg/g, consistent with recorded Merapi whole-rock values of 12 to 20 μg/g (ref. 31), but slightly lower than reported concentrations for Merapi clinopyroxene-hosted melt inclusions (35 to 109 μg/g; ref. 32).

Boron isotope profiles were analysed across the interface between CaO-rich and CaO-normal glass (Fig. 2). The δ^11^B values and B concentration of the CaO-normal glasses range from −5.3 to + 1.2‰ and from 10 to 15 μg/g for Merapi and from −14.7 to −4.9‰ and 9 to 241 μg/g for Vesuvius. In contrast, the δ^11^B values of the CaO-rich and (MgO)CaO-rich glasses range from −21.9 to −8.6‰ for Merapi and from −41.5 to −13.6‰ for Vesuvius. These values fall considerably below the δ^11^B values of many subduction systems globally and they are significantly lower than the experimental starting materials (Fig. 1d,e; see also Supplementary Table S5 for sources of all literature data presented). The δ^11^B values of the CaO-rich glasses are also considerably lower than those of carbonate in the literature. In general, biogenic carbonate has an average δ^11^B value of +19.1‰ and variable B concentration (Fig. 1). In contrast, lithified carbonate (*i.e.* lime/dolostone) has lower but still positive δ^11^B values, which range from +1.5 to +8.4‰ and B concentration of 2 to 18 μg/g (refs 33 and 34; Fig. 1, data sources in Supplementary Table S5). We therefore conclude that simple binary mixing between the reactants cannot explain the low δ^11^B melts in the experiments and implies additional processes at work.

Boron concentration in the CaO-rich and CaO(MgO)-rich glasses ranges from 4 to 7 μg/g and 2 to 85 μg/g for Merapi and Vesuvius, respectively. A negative correlation between δ^11^B value and B concentration is observed in all experiments (Fig. 1d,e) and hence the low δ^11^B values recorded must be related to B degassing from the melt(s) under the experimental conditions (see Methods). We also note that the experimental data (*n* = 172) for the most part overlap the range of natural magmatic values (Fig. 1), supporting the view that the experiments provide a useful analogue for natural magmatic processes. We hence argue that boron isotope fractionation during decarbonation in the upper plate represents another means to generate low δ^11^B values in arc magmas in addition to slab dehydration during subduction (*cf.* refs 3, 8, 9, 10 and 22).

As our experiments were heated to 1200 °C over a period of six minutes (see Methods), the fractionation factor between tetrahedrally and trigonally coordinated boron, α, varied with experiment duration, since α is inversely proportional to temperature^13^. Realising that α was not constant in our experiments precludes calculation of a unique Rayleigh model for our experimental data. To explain our lowest δ^11^B values, α probably varied from ca. 1.012 to 1.002 (the latter at 1200 °C), but these values have large uncertainties due to the fact that CO_2_ was continually fluxing in the experiments and that fractionation commenced already below 1200 °C. Nonetheless, a similar range of α values may apply to situations where a large thermal gradient exists, such as across metamorphic aureoles in plutonic complexes, or along down-going slabs in subduction zones. Boron isotope fractionation in the experimental melts would also have been mirrored by evolving δ^11^B values in the co-existing fluid, with δ^11^B values becoming lower over time in a similar fashion to the slab dehydration models performed by ref. 15. Assuming that boron isotope fractionation is independent of pressure^35^, our data are in-line with these and similar models that predict values lower than −30‰ in dehydrated subducted materials, and others as low as −35‰ for phengite-free dehydrated assemblages at subduction depths^15^. The fundamental implication here is that extremely low δ^11^B values can be generated in subducted material at ~3 GPa as well as in magma in the upper arc crust at ~0.5 GPa due to the presence of a coexisting fluid phase that serves to scavenge boron from the rock or silicate melt.

**Figure 3:**
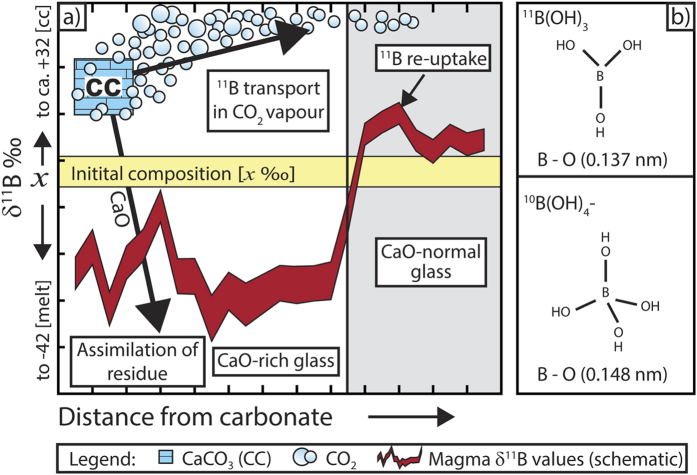
Conceptual model. Tetrahedrally coordinated boron is present in carbonate and silicate melt and decarbonation at the onset of assimilation triggers boron isotope fractionation as follows: CaCO_3_(BOH)_4_ (carbonate) +SiO_2_(BOH)_4_ (silicate melt) → CaO-rich silicate melt +^10^B(OH)_4_ (in melt) + CO_2_ (fluid) +^11^B(OH)_3_ (in CO_2_-rich fluid). In other words, assimilation of carbonate into the melt gives rise to Ca-rich melt and a co-existing CO_2_ phase that mingles with CaO-normal melt. Transport of trigonally coordinated ^11^B in CO_2_ bubbles away from the reaction site and subsequent partial reabsorption in CO_2_-undersaturated melt at the distal parts of the capsule gives rise to relatively high δ^11^B values in portions of the CaO-normal glass.

Our experiments also reveal several dynamic aspects of boron transport in magma. In particular, the relatively elevated δ^11^B values of some of the CaO-normal glass domains compared to the δ^11^B values of the starting material (Fig. 2) leads us to propose a conceptual model (Fig. 3). In our model, carbonate dissolution and degassing at the onset of magma-carbonate interaction is the catalyst for boron isotope fractionation. The newly formed CO_2_ phase scavenges ^11^B from the carbonate and silicate melt, causing ^11^B(OH)_3_ to enter the newly generated volatile phase by substitution for CO_2_ and assimilation of ^10^B-rich material to occur at the decarbonation reaction site. The highly mobile volatile phase then rapidly migrates away from the reaction site, resulting in the generation of a fluid with a relatively high δ^11^B value that progressively evolves towards lower δ^11^B values, similar to some arc fluids and models thereof, as discussed above (Fig. 1c). Conversely, the relatively unaffected, CaO-normal melt further away from the carbonate dissolution site would be undersaturated with respect to CO_2_, which could facilitate coupled ^11^B and CO_2_ reabsorption in the melt and hence relatively high δ^11^B melt values (Fig. 3). The extent to which this process is expressed as “low” or “high” δ^11^B melts in individual volcanic systems depends on several factors, including the amount of carbonate assimilated, the viscosity of the magma and, particularly, the solubility of CO_2_ in the melt, since low melt solubility of CO_2_ will promote bubble formation and thus boron extraction from the co-existing melt. This process would be most effective under low pressures, since CO_2_ solubility in silicate melts decreases with decreasing pressure (see discussion in refs 24, 25, 28 and references therein), which would make boron extraction into a CO_2_-bearing phase most efficient in the upper parts of the crust.

In conclusion, our data demonstrate that short time-scale (syn-eruptive) carbonate assimilation can result in heterogeneous and locally very low δ^11^B melt values, similar to predictions for subducted materials. Distinguishing between these processes may be aided by, *e.g.*, the presence or absence of crustal xenolith suites and by thermobarometry to constrain crystallisation depth of the main mineral phases. Boron isotope fractionation in magma via crustal carbonate dissolution is likely to be most pertinent for volcanoes sited on volatile-bearing sedimentary crust, as for instance found in continental subduction settings, and may help to identify upper crustal additions to the carbon cycle.

## Methods

### Experimental methods

The experiments presented in this paper were designed to simulate assimilation of carbonate crust by magma using Merapi and Vesuvius volcanoes as type examples. All experiments were carried out using the end-loaded piston cylinder apparatus at the HP-HT Laboratory of Experimental Volcanology and Geophysics, at the Istituto Nazionale di Geofisica e Vulcanologia (INGV), Rome, Italy (see http://www.roma1.ingv.it/laboratori/laboratorio-hp-ht/). The end-loaded piston cylinder is calibrated for use in the pressure range 0.5–2 GPa (±50 MPa) and the lowest end of this range was selected for the experiments (0.5 GPa), which corresponds to approximately mid-crustal depths. The experiments were carried out under super-liquidus conditions for the magmatic starting material by employing an experimental temperature of 1200 °C (±5 °C).

The following starting materials were used in the experiments (see Supplementary Table S1):

*(i) Merapi series*. A sample of 1994 Merapi basaltic-andesite whole rock powder was fused and hydrated with ultrapure Milli-Q water to ca. 2.5 wt. % H_2_O. This glass sample was powdered again for insertion into the experimental capsules. The crustal carbonate reactant used in the Merapi series was a sample of local Java platform carbonate, sourced from a quarry to the south of Merapi volcano. The carbonate was inserted into the capsules as small, solid fragments of rock, weighing ~9 to 10 mg. Petrographic descriptions and major element compositions of the starting materials used in the Merapi series are provided in ref. 24.

*(ii) Vesuvius series*. The starting materials used in the Vesuvius experiments were a shoshonitic lava flow from Vesuvius^29^ fused and hydrated with ultrapure Milli-Q water to ca. 2.0 wt. % H_2_O and limestone and dolostone from the local Procida carbonate formation^36^,^37^. The limestone and dolostone added to the experimental charges were small solid fragments of rock, weighing ~6 to 8 mg. Two sets of experiments were performed containing a limestone and shoshonite hydrated glass and a dolostone and shoshonitic hydrated glass. Detailed descriptions and compositions of the experimental products are provided in ref. 25.

The experiment starting materials were loaded into platinum capsules with 3 mm outer diameter before insertion into a 19.1mm NaCl–crushable alumina–pyrophyllite–pyrex assembly^38^. The capsules were pressurised to 0.5 GPa and then heated from ambient temperature to the target temperature of 1200 °C using a ca. 6 min duration heat-up ramp. Thereafter, the experiments were either i) quenched immediately on reaching the target temperature; these experiments constituted “zero-time” runs; or ii) held at the target temperature for durations of 60 s, 90 s, 150 s (Merapi only), and 300 s before quenching. A detailed description of the experimental method is provided in refs 24 and 25 and references therein.

At the end of the runs, the capsules were retrieved from the piston cylinder and mounted in low-volatility Struers EpoFix epoxy resin under vacuum to impregnate the void spaces and prevent sample loss that may otherwise occur due to the high vesicularity of the samples. The long axis of the capsules were placed parallel to the surface of the epoxy block and the capsules were subsequently polished along their long axes until the enclosed experimental products were revealed on the surface at a depth corresponding to approximately the middle of the capsule thickness. The samples were then carbon coated for electron microprobe (EMP) imaging and analysis at INGV Rome. Representative analyses of the CaO-normal and (MgO)CaO-rich experimental glasses are given in Supplementary Table S2. Prior to SIMS analysis, the carbon coating was gently removed from the experiments by polishing with 1 μm diamond-paste, after which the samples were cleaned with pure ethanol and coated with gold for Secondary Ionisation Mass Spectrometry (SIMS) analysis.

### SIMS protocol

Boron isotope analyses were performed using the CAMECA IMS 1280 ion microprobe at the Nordsim facility in Stockholm, Sweden, employing an analytical protocol based on ref. 39. An O_2_^−^ primary beam of 6 nA with an accelerating voltage of 13.0 kV and imaging a 200 μm aperture was used to produce a 20 μm analysis spot. The primary beam was employed to sputter a 25 μm square raster for 150 seconds prior to data acquisition to eliminate surface contamination. Secondary ions were transferred to the mass spectrometer using a nominal potential of 10 kV and measured at a mass resolution (M/ΔM) of 2860, which is sufficient to eliminate isobaric interference of ^10^B^1^H^+^ on ^11^B. Signals were measured by magnet peak switching into an ion counting electron multiplier, and the sequence comprised a measurement at mass 9.33 (2 s integration time), ^10^B^+^ (8 s), ^11^B^+^ (4 s), and ^30^Si^++^ (2 s). Instrumental mass fractionation was determined and corrected for by employing a reference volcanic glass from Lipari Island (B6) with δ^11^B_NBS_ and B concentration values of −3.3‰ and 197 μg/g, respectively^34^. We note that in an inter-laboratory comparison study, the δ^11^B values of B6 showed a large degree of variance of ±3.6‰ (2 SD)^34^. Despite this, B6 behaves extremely well on the micron-scale, as it is homogeneous and boron-rich. The value chosen here of −3.3‰ for B6 is based on analyses by G.D. Layne (pers. comm.) calibrated to reference material GB-4 that has an accepted value of −12.8‰ (*e.g.*, ref. 40). This value of −3.3‰ also corresponds to one of the values obtained by positive thermal ionisation mass spectrometry (PTIMS) in the inter-laboratory comparison study^34^. As our experimental and starting material glasses were both analysed using the same method and employing the same value for standard B6 (−3.3‰), our data are internally consistent and the relative enrichment and/or depletion patterns in our boron isotope data are valid. However, there may be a systematic bias in our data when compared to data generated by different SIMS laboratories using different values for the B6 glass standard.

The ^11^B yields ranged from 500 to 700 cps/μg g^−1^/nA for the standard. Internal precision (1σ mean) on ^11^B/^10^B ranged from ±0.5‰ to ±2.9‰ (se_mean_) for both B6 and the unknowns. The data were acquired over several analytical sessions, with the external error (reproducibility) on B6 ranging from ±0.42 to 1.29‰ (RSD; *n* = 69). External errors were propagated onto the overall analytical uncertainty for each analysis. The uncertainty on the boron concentration measurements (determined from ^10^B/^30^Si^++^ ratios) is estimated at 4.3% (RSD) which, when propagated with the internal errors, gives an absolute uncertainty of ca. 10% (RSD) at the 2σ level. Boron data for the fused starting materials (*n* = 25) and the experimental products (*n* = 147) are provided in Supplementary Tables S3 and S4.

## Additional Information

**How to cite this article**: Deegan, F. M. *et al*. Boron isotope fractionation in magma via crustal carbonate dissolution. *Sci. Rep.* **6**, 30774; doi: 10.1038/srep30774 (2016).

## Supplementary Material

Supplementary Information
