# Supplementary material for: Boron isotope fractionation in magma via crustal carbonate dissolution
Source: Sci Rep. 2016 Aug 4;6:30774. doi: 10.1038/srep30774 (PMC4973271; doi:10.1038/srep30774)
Supplement: Supplementary Information [file srep30774-s1.pdf]

Boron isotope fractionation in magma via crustal carbonate dissolution

Frances M. Deegan, Valentin R. Troll, Martin J. Whitehouse, Ester M. Jolis, Carmela Freda

Supplementary Information

Contents:

Table S1 Major element composition of Merapi and Vesuvius starting materials used in experiments

Table S2 Representative major element composition of Merapi and Vesuvius experimental glasses (CaO-normal and (MgO)CaO-rich)

Table S3 Boron concentration and isotope data for the starting magmatic glasses used in magma-carbonate interaction experiments

Table S4 Boron concentration and isotope data for magma-carbonate interaction experimental glasses

Table S5 Boron concentration and isotope data for selected geochemical reservoirs from the literature (plotted in Figure 1)

References cited in Supplementary Information

Table S1. Major element composition of Merapi and Vesuvius starting materials used in experiments

	Average composition of hydrous Merapi glass[1,2]	1σ (9)[3]	Average composition of hydrous Vesuvius glass[1,2]	1σ (9)[3]	Whole rock analysis of Merapi limestone added[4]	Whole rock analysis of Vesuvius limestone added[2]	Whole rock analysis of Vesuvius dolostone added[2]
wt%							
SiO_2	51.83	0.43	49.85	0.21	0.28	0.02	0.02
TiO_2	0.89	0.05	1.03	0.08	0.01	-	-
Al_2O_3	18.08	0.24	15.95	0.67	0.13	-	-
FeO_t^*	8.17	0.16	7.98	0.25	0.01	0.01	0.00
MnO	0.20	0.03	0.14	0.02	0.00	0.01	0.01
MgO	2.97	0.08	6.03	0.33	0.40	0.79	21.01
CaO	9.19	0.16	9.98	0.29	56.72	54.99	31.34
Na_2O	3.48	0.09	2.35	0.07	0.12	-	-
K_2O	2.05	0.03	4.01	0.19	0.00	-	-
P_2O_5	0.34	0.04	0.70	0.09	0.03	0.02	0.00
Total	97.20		98.29		57.70	55.84	52.38
H_2O	2.23		1.71		0.15	-	-
CO_2	-		-		44.93	44.11	47.59

[1] Merapi hydrous glass was uniformly hydrated at Universität Hannover (Germany) and measured for water content by Karl Fischer Titration (KFT). Vesuvius hydrous glass was uniformly hydrated at INGV (Italy) and the water content estimated by the difference between the total and 100 wt.%.
[2] Analysis carried out by EMP at INGV (Rome, Italy).
[3] 1σ standard deviation. The number in parentheses represents the number of analyses for each sample.
[4] XRF analysis carried out at IFM-GEOMAR (Kiel, Germany).
[*] Total iron is given as a FeO_t.
Data sources: Deegan *et al.* (2010) and Jolis *et al.* (2013).

Table S2. Representative major element composition of Merapi and Vesuvius experimental glasses (CaO-normal and (MgO)CaO-rich)

	CaO-normal glass					(MgO)CaO-rich glass				
Run-sample	375-7[1]	374-5[1]	V4[2]	V9[2]	V1[2]	375-7[1]	374-5[1]	V4[2]	V9[2]	V1[2]
wt%										
SiO_2	51.83	50.88	49.15	49.21	50.31	35.99	38.11	37.02	24.81	28.77
TiO_2	0.83	0.89	0.93	0.96	0.99	0.44	0.58	0.72	0.66	0.51
Al_2O_3	18.22	18.72	16.61	15.65	15.50	13.04	13.94	12.12	7.93	9.07
FeO_t*	7.50	6.24	6.58	6.79	6.82	5.85	5.48	5.39	4.49	5.07
MnO	0.22	0.20	0.12	0.14	0.15	0.17	0.14	0.06	0.13	0.12
MgO	2.69	2.73	5.70	5.62	5.96	2.25	2.26	9.04	3.97	4.49
CaO	9.16	8.84	9.14	10.00	9.74	29.77	27.41	20.75	34.99	35.29
Na_2O	4.25	4.48	3.15	2.65	2.51	1.90	2.22	2.41	1.10	1.16
K_2O	2.32	2.26	4.05	4.92	4.28	1.07	1.21	2.31	1.45	1.28
P_2O_5	0.36	0.27	0.58	0.64	0.62	0.25	0.22	0.50	0.38	0.37
Total**	97.38	95.52	96.01	96.76	96.93	90.73	91.58	90.42	80.00	86.24

[1] Merapi series: 375-7, limestone reactant, 150 s duration; 374-5, limestone reactant, 300s duration.
[2] Vesuvius series: V4, dolostone reactant, 60 s duration; V9, limestone reactant, 60 s duration; V1, limestone reactant, 90 s duration.
*Total iron is given as a FeO_t.
**Analysis totals are low due to *a)* microbubbles (cf. Deegan *et al*., 2010), *b)* increased CO_2 solubility in alkaline and ultrapotassic melts in general (Behrens, 1995) or *c)* increased CO_2 solubility in the CaO-rich experimental melt (Moore, 2008).
Data sources: Deegan *et al.* (2010) and Jolis *et al.* (2013).

Table S3. Boron concentration and isotope data for the starting magmatic glasses used in magma-carbonate interaction experiments

SIMS I.D.	B ppm	$\delta^{11}B$ ‰	± ‰
Merapi starting glass			
1141_M94-26_1@1	17	-8.4	1.7
1141_M94-26_2@1	17	-7.0	1.6
1141_M94-26_3@1	15	-7.0	1.6
1141_M94-26_4@1	16	-7.6	1.7
1141_M94-26_5@1	18	-6.8	1.8
1141_M94-26_6@1	18	-5.6	1.7
1141_M94-26_7@1	18	-4.5	1.8
1141_M94-26_5@2	17	-4.9	2.2
1141_M94-26_3@2	16	-8.8	1.6
1141_M94-26_8@1	18	-4.3	1.6
1141_M94-26_2@2	16	-4.6	1.6
1141_M94-26_1@2	18	-3.5	1.6
Vesuvius starting glass			
1141_PC428_1@1	13	-11.8	1.8
1141_PC428_2@1	13	-12.3	1.6
1141_PC428_3@1	13	-13.9	1.5
1141_PC428_4@1	13	-13.0	1.5
1141_PC428_5@1	13	-13.9	1.6
1141_PC428_1@2	12	-7.6	1.5
1141_PC428_6@1	13	-10.7	1.5
1141_PC428_7@1	13	-10.1	1.6
1141_PC428_8@1	14	-12.5	1.5
1141_PC428_5@2	13	-11.3	1.6
1141_PC428_3@2	13	-14.2	1.5
1141_PC428_9@1	13	-12.3	1.6
1141_PC428_9@2	13	-14.6	1.6

Table S4. Boron concentration and isotope data for magma-carbonate interaction experimental glasses

Experiment I.D.	SIMS I.D.	Glass type	B ppm	$\delta^{11}B$ ‰	± ‰
Vesuvius series					
V4, dolostone reactant, 1200°C, 0.5 GPa, 60 s duration					
V4	V4-trav1-1	CaO-normal	72	-7.9	0.6
V4	V4-trav1-2	(MgO)CaO-rich	53	-14.6	0.8
V4	V4-trav1-3	(MgO)CaO-rich	51	-13.6	0.7
V4	V4-trav1-4	(MgO)CaO-rich	30	-20.9	0.8
V4	V4-trav1-5	(MgO)CaO-rich	29	-22.4	0.8
V4	V4-trav1-6	CaO-normal	76	-9.3	0.7
V4	V4-trav1-7	(MgO)CaO-rich	41	-14.6	0.8
V9, limestone reactant, 1200°C, 0.5 GPa, 60 s duration					
V9	V9-trav1-1	CaO-normal	18	-12.1	1.0
V9	V9-trav1-2	CaO-normal	18	-12.9	1.0
V9	V9-trav1-3	CaO-normal	16	-14.3	1.1
V9	V9-trav1-4	CaO-normal	16	-10.5	1.1
V9	V9-trav1-5	CaO-normal	15	-12.4	1.1
V9	V9-trav1-6	CaO-normal	15	-11.5	1.2
V9	V9-trav1-7	CaO-normal	15	-10.0	1.2
V9	V9-trav1-8	CaO-normal	16	-11.6	1.1
V9	V9-trav1-9	CaO-normal	15	-12.0	1.1
V9	V9-trav1-11	CaO-normal	15	-10.9	1.1
V9	V9-trav1-12	CaO-normal	14	-11.4	1.1
V9	V9-trav1-13	CaO-normal	14	-9.3	1.1
V9	V9-trav1-14	CaO-normal	14	-10.9	1.2
V9	V9-trav1-15	CaO-normal	14	-8.2	1.1
V9	V9-trav1-16	CaO-normal	13	-13.0	1.2
V9	V9-trav1-17	CaO-normal	12	-10.6	1.3
V9	V9-trav1-18	CaO-normal	12	-14.4	1.2
V9	V9-trav1-19	CaO-normal	9	-14.7	1.4
V9	V9-trav1-20	CaO-normal	11	-12.3	1.3
V9	V9-trav1-21	CaO-rich	2	-37.1	2.6
V9	V9-trav1-22	CaO-rich	3	-40.5	2.9
V9	V9-trav1-23	CaO-rich	5	-24.3	1.9
V9	V9-trav1-24	CaO-rich	3	-32.6	2.2
V9	V9-trav1-25	CaO-rich	3	-37.8	2.4
V9	V9-trav1-26	CaO-rich	4	-25.8	1.9
V9	V9-trav1-27	CaO-rich	4	-29.7	2.0
V9	V9-trav1-29	CaO-rich	4	-24.9	2.1
V9	V9-trav1-30	CaO-rich	2	-39.6	2.6
V9	V9-trav1-31	CaO-rich	3	-31.8	2.2
V9	V9-trav1-32	CaO-rich	3	-35.5	2.4
V9	V9-trav1-33	CaO-rich	3	-37.8	2.4
V9	V9-trav1-35	CaO-rich	3	-33.8	2.3
V9	V9-trav1-36	CaO-rich	4	-26.3	2.0
V9	V9-trav1-37	CaO-rich	3	-24.2	2.2
V9	V9-trav1-38	CaO-rich	4	-25.9	2.0
V9	V9-trav1-39	CaO-rich	4	-28.4	2.1
V9	V9-trav1-41	CaO-rich	7	-17.1	1.6

Table S4 continued.

Experiment I.D.	SIMS I.D.	Glass type	B ppm	$\delta^{11}B$ ‰	± ‰
V1, limestone reactant, 1200°C, 0.5 GPa, 90 s duration					
V1	V1-trav1-1	CaO-normal	161	-6.3	0.8
V1	V1-trav1-2	CaO-normal	163	-5.6	0.6
V1	V1-trav1-3	CaO-normal	166	-6.6	0.6
V1	V1-trav1-6	CaO-normal	237	-6.2	0.6
V1	V1-trav1-7	CaO-normal	240	-7.5	0.6
V1	V1-trav1-8	CaO-normal	141	-11.7	0.6
V1	V1-trav1-9	CaO-normal	177	-8.5	0.6
V1	V1-trav1-10	CaO-normal	195	-8.8	0.6
V1	V1-trav1-12	CaO-normal	133	-10.1	0.6
V1	V1-trav1-13	CaO-rich	70.8	-24.9	0.6
V1	V1-trav1-14	CaO-rich	85	-22.3	0.7
V1	V1-trav1-15	CaO-rich	64	-25.5	0.7
V1	V1-trav1-16	CaO-rich	60	-21.8	0.7
V1	V1-trav1-18	CaO-rich	46	-29.6	0.8
V1	V1-trav1-19	CaO-rich	66	-23.2	0.7
V1	V1-trav1-20	CaO-normal	241	-10.1	0.6
V1	V1-trav1-21	CaO-normal	166	-9.2	0.6
V1	V1-trav1-22	CaO-normal	163	-9.6	0.7
V1	V1-trav2-2	CaO-normal	226	-7.6	0.6
V1	V1-trav2-3	CaO-normal	128	-9.8	0.7
V1	V1-trav2-4	CaO-rich	51	-22.2	0.8
V1	V1-trav2-5	CaO-rich	51	-20.2	0.8
V1	V1-trav2-6	CaO-rich	38	-27.9	0.9
V1	V1-trav2-8	CaO-rich	52	-25.1	0.8
V1	V1-trav2-9	CaO-rich	43	-27.2	0.7
V1	V1-trav2-10	CaO-rich	54	-23.7	0.7
V1	V1-trav2-11	CaO-rich	56	-21.6	0.7
V1	V1-trav2-12	CaO-rich	80	-19.8	0.7
V1	V1-trav2-14	CaO-normal	155	-9.1	0.6
V1	V1-trav2-15	CaO-normal	144	-8.1	0.6
V1	V1-trav2-16	CaO-normal	160	-6.9	0.6
V1	V1-trav2-17	CaO-normal	180	-6.3	0.6
V1	V1-trav2-18	CaO-normal	154	-7.1	0.6
V1	V1-trav3-1	CaO-normal	163	-7.2	0.6
V1	V1-trav3-2	CaO-normal	181	-6.4	0.6
V1	V1-trav3-3	CaO-normal	268	-4.9	0.6
V1	V1-trav3-4	CaO-normal	189	-5.8	0.6
V1	V1-trav3-6	CaO-normal	160	-6.3	0.6
Merapi series					
375-7, limestone reactant, 1200°C, 0.5 GPa, 150 s duration					
375-7	375-7trav2-2	CaO-rich	6	-17.1	1.7
375-7	375-7trav2-3	CaO-rich	5	-14.3	1.9
375-7	375-7trav2-4	CaO-rich	5	-20.9	1.8
375-7	375-7trav2-5	CaO-rich	5	-15.9	1.8
375-7	375-7trav2-6	CaO-rich	6	-14.0	1.7
375-7	375-7trav2-7	CaO-rich	7	-9.9	1.7

Table S4 continued.

Experiment I.D.	SIMS I.D.	Glass type	B ppm	$\delta^{11}B$ ‰	± ‰
375-7	375-7trav2-8	CaO-rich	5	-17.4	1.9
375-7	375-7trav2-9	CaO-rich	4	-17.4	1.9
375-7	375-7trav2-10	CaO-rich	4	-21.9	2.5
375-7	375-7trav2-11	CaO-rich	4	-19.0	2.0
375-7	375-7trav2-12	CaO-rich	4	-20.4	2.3
375-7	375-7trav2-13	CaO-rich	4	-19.4	2.0
375-7	375-7trav2-14	CaO-rich	5	-18.1	1.9
375-7	375-7trav2-15	CaO-rich	4	-18.3	2.0
375-7	375-7trav2-16	CaO-rich	5	-18.2	1.9
375-7	375-7trav2-17	CaO-rich	5	-17.9	2.0
375-7	375-7trav2-18	CaO-rich	7	-11.0	1.8
375-7	375-7trav2-19	CaO-normal	13	-2.2	1.2
375-7	375-7trav2-20	CaO-normal	14	-0.9	1.2
375-7	375-7trav2-21	CaO-normal	14	+0.1	1.2
375-7	375-7trav2-22	CaO-normal	14	-2.8	1.1
375-7	375-7trav2-23	CaO-normal	14	-4.0	1.3
375-7	375-7trav2-24	CaO-normal	14	-2.3	1.2
375-7	375-7trav2-25	CaO-normal	14	-3.3	1.2
375-7	375-7trav2-26	CaO-normal	14	-2.8	1.1
375-7	375-7trav3-1	CaO-rich	7	-9.8	1.5
375-7	375-7trav3-2	CaO-rich	4	-22.2	2.1
375-7	375-7trav3-3	CaO-rich	4	-18.8	2.2
375-7	375-7trav3-4	CaO-rich	4	-20.9	2.0
375-7	375-7trav3-5	CaO-rich	4	-21.6	2.0
375-7	375-7trav3-6	CaO-normal	10	-5.3	1.3
375-7	375-7trav3-7	CaO-normal	13	-3.3	1.4
375-7	375-7trav3-8	CaO-normal	13	-3.1	1.2
375-7	375-7trav3-9	CaO-normal	14	-2.8	1.2
375-7	375-7trav3-10	CaO-normal	13	-1.8	1.2
375-7	375-7trav3-11	CaO-normal	14	-3.7	1.2
375-7	375-7trav3-12	CaO-normal	14	-3.7	1.2
375-7	375-7trav3-13	CaO-normal	13	-3.6	1.2
375-7	375-7trav3-14	CaO-normal	13	-3.0	1.2
375-7	375-7trav3-15	CaO-normal	13	-3.9	1.3
374-5, limestone reactant, 1200°C, 0.5 GPa, 300s duration					
374-5	374-5trav1-1	CaO-normal	12	-3.1	1.3
374-5	374-5trav1-3	CaO-rich	9	-9.5	1.4
374-5	374-5trav1-4	CaO-rich	10	-10.0	1.6
374-5	374-5trav1-5	CaO-rich	10	-9.7	1.4
374-5	374-5trav1-6	CaO-rich	7	-10.6	2.4
374-5	374-5trav1-7	CaO-rich	6	-16.2	1.7
374-5	374-5trav1-8	CaO-rich	10	-10.4	1.4
374-5	374-5trav1-9	CaO-rich	6	-16.5	1.8
374-5	374-5trav1-10	CaO-rich	6	-16.6	1.7
374-5	374-5trav1-11	CaO-rich	6	-9.8	1.7
374-5	374-5trav1-12	CaO-rich	6	-13.7	1.9
374-5	374-5trav1-13	CaO-rich	6	-16.9	1.8

Table S4 continued.

Experiment I.D.	SIMS I.D.	Glass type	B ppm	$\delta^{11}B$ ‰	± ‰
374-5	374-5trav1-14	CaO-rich	7	-11.1	1.7
374-5	374-5trav1-15	CaO-normal	12	-2.8	1.3
374-5	374-5trav1-16	CaO-rich	6	-12.5	1.7
374-5	374-5trav1-17	CaO-rich	7	-14.0	1.6
374-5	374-5trav1-18	CaO-rich	7	-13.7	1.7
374-5	374-5trav1-19	CaO-normal	13	-4.4	1.2
374-5	374-5trav1-20	CaO-normal	13	-1.0	1.2
374-5	374-5trav1-21	CaO-normal	13	-0.6	1.2
374-5	374-5trav1-22	CaO-normal	13	-2.2	1.2
374-5	374-5trav1-23	CaO-normal	13	0.0	1.2
374-5	374-5trav1-24	CaO-normal	13	-0.8	1.2
374-5	374-5trav1-25	CaO-normal	13	+1.2	1.3

All data obtained using a CAMECA IMS 1280 SIMS instrument at the Swedish Museum of Natural History in Stockholm (Nordsim).

Table S5. Boron concentration and isotope data for selected geochemical reservoirs from the literature (plotted in Figure 1)

	B ppm range	$\delta^{11}B$ ‰ range	Reference
Surface, slab, mantle, and arc-related reservoirs			
Seawater	4 to 5	+39.3 to +40.2	Spivack and Edmond, 1987; Hemming and Hanson, 1992; Foster *et al*., 2010
Carbonates (sediments, biogenic skeletons, corals, foraminifera, calcite)	4 to 58	+8.9 to +39.8	Hemming and Hanson, 1992; Vengosh *et al*., 1991; Spivack *et al*., 1993; Gaillardet and Allègre, 1995; Deyhle *et al*., 2001
Lime- and dolostone	2 to 18	+1.5 to +8.4	Vengosh *et al*., 1991; Gonfiantini *et al*., 2003
Altered ocean crust (AOC)	1 to 104	-4.3 to +24.9	Smith *et al*., 1995
Marine sediment	13 to 163	-17.0 to +10.5	Ishikawa and Nakamura, 1993; Moriguti and Nakamura, 1998; Tonarini *et al*., 2011
Mid Ocean Ridge Basalt (MORB)	0.3 to 3.6	-10.8 to -1.2	Spivack and Edmond, 1987; Chaussidon and Jambon, 1994; Moriguti and Nakamura, 1998; le Roux *et al*., 2004; Ganoun *et al*., 2007; Shaw *et al*., 2012; Marschall and Monteleone, 2014
Ocean Island Basalt (OIB)	0.3 to 7.8	-16.9 to +7.8	Chaussidon and Jambon, 1994; Chaussidon and Marty, 1995; Gurenko and Chaussidon, 1997; Kobayashi *et al*., 2004; Turner *et al*., 2007; Brounce *et al*., 2012
Arc metamorphic rocks and minerals	0.4 to 59	-15.6 to +7.9	Peacock and Hervig, 1999; Nakano and Nakamura, 2001; Pabst *et al*., 2012
Island arcs			
Halmahera	2 to 32	-2.3 to +3.6	Palmer, 1991
Izu	3 to 65	-6.8 to +12.0	Ishikawa and Nakamura, 1994; Straub and Layne, 2002
Kurile	5 to 48	-3.8 to +5.9	Ishikawa and Tera, 1997
Lesser Antilles	2 to 102	-25.6 to +12.9	Smith *et al*., 1997; Bouvier *et al*., 2008; Bouvier *et al*., 2010
Manus		-0.6 to +5.8	Shaw *et al*., 2012
Marianas	9 to 28	+2.9 to +6.2	Ishikawa and Tera, 1999
NE Japan	3 to 15	-4.7 to +3.4	Moriguti *et al*., 2004
S. Sandwich	3 to 20	+12.1 to +17.6	Tonarini *et al*., 2011
Aeolian Islands	5 to 60	-13.7 to +2.3	Tonarini *et al*., 2001a; Schiavi *et al*., 2012

	B ppm range	$\delta^{11}B$ ‰ range	Reference
Continental arcs and volcanoes			
Anatolia	7 to 66	-15.1 to -0.2	Tonarini *et al.*, 2005
Andes	10 to 60	-7.2 to +4.2	Rosner *et al.*, 2003
Cascades	1 to 18	-21.3 to +4.4	Rose *et al.*, 2001; Leeman *et al.*, 2004; Le Voyer et al., 2010
Ecuadorian Andes	6 to 12	-17.9 to +3.5	Le Voyer *et al.*, 2008
El Salvador	5 to 36	-2.7 to +6.3	Tonarini *et al.*, 2007
Kamchatka	11 to 36	-3.7 to +5.6	Ishikawa *et al.*, 2001
Phlegrean province	5 to 118	-10.6 to -2.8	Tonarini *et al.*, 2004; 2009
Vesuvius and Etna	7 to 36	-7.6 to -3.5	Tonarini *et al.*, 2001b; Di Renzo *et al.*, 2007

References cited in Supplementary Information

Behrens, H. (1995) Determination of water solubilities in high-viscosity melts: An experimental study on $NaAlSi_3O_8$ and $KAlSi_3O_8$ melts. *European Journal of Mineralogy* 7, 905-920.

Bouvier, A.-S., Métrich, N., Deloule, E. (2008) Slab-derived fluids in the magma sources of St. Vincent (Lesser Antilles arc): volatile and light element imprints. *Journal of Petrology* 49, 1427-1448.

Bouvier, A.-S., Métrich, N., Deloule, E. (2010) Light elements, volatiles, and stable isotopes in basaltic melt inclusions from Grenada, Lesser Antilles: inferences for magma genesis. *Geochemistry Geophysics Geosyst*ems 8, Q09004.

Brounce, M., Feineman, M., LaFemina, P., Gurenko, A. (2012) Insights into crustal assimilation by Icelandic basalts from boron isotopes in melt inclusions from the 1783-1784 Lakagígar eruption. *Geochimica et Cosmochimica Acta* 94, 164-180.

Chaussidon, M., Jambon, A. (1994) Boron content and isotopic composition of oceanic basalts: geochemical and cosmochemical implications. *Earth and Planetary Science Letters* 121, 277-291.

Chaussidon, M., Marty, B. (1995) Primitive boron isotope composition of the mantle. *Science* 269, 383-386.

Deegan, F.M., Troll, V.R., Freda, C., Misiti, V., Chadwick, J.P., McLeod, C.L., Davidson, J.P. (2010) Magma-carbonate interaction processes and associated CO_2 release at Merapi volcano, Indonesia: insights from experimental petrology. *Journal of Petrology* 51, 1027–1051.

Deyhle, A., Kopf, A., Eisenhauer, A. (2001) Boron systematics of authigenic carbonates: a new approach to identify fluid processes in accretionary prisms. *Earth and Planetary Science Letters* 187, 191-205.

Di Renzo, V., Di Vito, M.A., Arienzo, I., Carandente, A., Civetta, L., D'Antonio, M., Giordano, F., Orsi, G., Tonarini, S. (2007) Magmatic History of Somma-Vesuvius on the basis of new

geochemical and isotopic data from a deep borehole (Camaldoli della Torre). *Journal of Petrology* 48, 753–784.

Foster, G.L., Pogge von Strandmann, P.A.E., Rae, J.W.B. (2010) Boron and magnesium isotopic composition of seawater. *Geochemistry Geophysics Geosyst*ems 11, Q08015.

Gaillardet, J., Allègre, C.J. (1995) Boron isotopic compositions of corals: seawater or diageneis record? *Earth and Planetary Science Letters* 136, 665-676.

Gannoun, A., Burton, K.W., Parkinson, I.J., Alard, O., Sciano, P., Thomas, L.E. (2007) The scale and origin of the osmium isotope variations in mid-ocean ridge basalts. *Earth and Planetary Science Letters* 259, 541-556.

Gonfiantini, R., Tonarini, S., Gröning, M., Adorni-Braccesi, A., Al-Ammar, A.S., Astner, M., Bächler, S., Barnes, R.M., Bassett, R.L., Cocherie, A., Deyhle, A., Dini, A., Ferrara, G., Gaillardet, J., Grimm, J., Guerrot, C., Krähenbühl, U., Layne, G., Lemarchand, D., Meixner, A., Northington, D.J., Pennisi, M., Reitznerová, E., Rodushkin, I., Sugiura, N., Surberg, R., Tonn, S., Wiedenbeck, M., Wunderli, S., Xiao, Y., Zack, T. (2003) Intercomparison of boron isotope and concentration measurements. Part II: evaluation of results. *Geostandards and Geoanalytical Research* 27, 41–57.

Gurenko, A.A., Chuassidon, M. (1997) Boron concentrations and isotopic composition of the Icelandic mantle: evidence from glass inclusions in olivine. *Chemical Geology* 135, 21-34.

Hemming, N.G., Hanson, G.N. (1992) Boron isotopic composition and concentration in modern marine carbonates. *Geochimica et Cosmochimica Acta* 56, 537-543.

Ishikawa, T., Nakamura, E. (1993) Boron isotope systematics of marine sediments. *Earth and Planetary Science Letters* 117, 567-580.

Ishikawa, T., Nakamura, E. (1994) Origin of the slab component in arc lavas from across-arc variation of B and Pb isotopes. *Nature* 370, 205-208.

Ishikawa, T., Tera, F. (1997) Source, composition and distribution of the fluid in the Kurile mantle wedge: constraints from across-arc variations of B/Nb and B isotopes. *Earth and Planetary Science Letters* 152, 123-138.

Ishikawa, T., Tera, F. (1999) Two isotopically distinct fluid components involved in the Mariana arc: evidence from Nb/B ratios and B, Sr, Nd, and Pb isotope systematics. *Geology* 27, 83-86.

Ishikawa, T., Tera, F., Nakazawa, T. (2001) Boron isotope and trace element systematics of the three volcanic zones in the Kamchatka arc. *Geochimica et Cosmochimica Acta* 65, 4523-4537.

Jolis, E.M., Freda, C., Troll, V.R., Deegan, F.M., Blythe, L.S., McLeod, C.L., Davidson, J.P. (2013) Experimental simulation of magma–carbonate interaction beneath Mt. Vesuvius, Italy. *Contributions to Mineralogy and Petrology* 166, 1335–1353.

Kobayashi, K., Tanaka, R., Moriguti, T., Shimizu, K., Nakamura, E. (2004) Lithium, boron, and lead isotope systematics of glass inclusions in olivines from Hawaiian lavas: evidence for recycled components in the Hawaiian plume. *Chemical Geology* 212, 143-161.

Leeman, W.P., Tonarini, S., Chan, L.H., Borg, L.E. (2004) Boron and lithium isotopic variations in a hot subduction zone-the southern Washington Cascades. *Chemical Geology* 212, 101-124.

le Roux, P.J., Shirey, S.B., Benton, L., Hauri, E.H., Mock, T.D. (2004) In situ, multiple-multiplier, laser ablation ICP-MS measurement of boron isotopic composition ($\delta^{11}B$) at the nanogram level. *Chemical Geology* 203, 123-138.

le Voyer, M., Rose-Koga, E.F., Laubier, M., Schiano, P. (2008) Petrogenesis of arc lavas from the Rucu Pichincha and Pan de Azucar volcanoes (Ecuadorian arc): major, trace element, and

boron isotope evidences from olivine-hosted melt inclusions. *Geochemistry Geophysics Geosystems* 9, Q12027.

Le Voyer, M., Rose-Koga, E.F., Shimizu, N., Grove, T.L., Schiano, P. (2010) Two contrasting H_2O-rich components in primary melt inclusions from Mount Shasta. *Journal of Petrology* 51, 1571-1595.

Marschall, H.R., Monteleone, B.D. (2014) Boron isotope analysis of silicate glass with very low boron concentrations by secondary ion mass spectrometry. *Geostandards and Geoanalytical Research* 39, 31-46.

Moore, G. (2008) Interpreting H_2O and CO_2 contents in melt inclusions: constraints from solubility experiments and modeling. *Reviews in Mineralogy and Geochemistry* 69, 333-362.

Moriguti, T., Nakamura, E. (1998) Across-arc variation of Li isotopes in lavas and implications for crust/mantle recycling at subduction zones. *Earth and Planetary Science Letters* 163, 167-174.

Moriguti, T., Shibata, T., Nakamura, E. (2004) Lithium, boron and lead isotope and trace element systematics of Quaternary basaltic volcanic rocks in northeastern Japan: mineralogical controls on salb-derived fluid composition. *Chemical Geology* 212, 81-100.

Nakano, T., Nakamura, E. (2001) Boron isotope geochemistry of metasedimentary rocks and tourmalines in a subduction zone metamorphic suite. *Physics of the Earth and Planetary Interiors* 127, 233-252.

Pabst, S., Zack, T., Savov, I.P., Ludwig, T., Rost, D., Tonarini, S., Vicenzi, E.P. (2012) The fate of subducted oceanic slabs in the shallow mantle: insights from boron isotopes and light element composition of metasomatized blueschists from the Mariana forearc. *Lithos* 132-133, 162–179.

Palmer, M.R. (1991) Boron-isotope systematics of Halmahera arc (Indonesia) lavas: evidence for involvement of the subducted slab. *Geology* 19, 215-217.

Peacock, S.M., Hervig, R.L. (1999) Boron isotopic composition of subduction-zone metamorphic rocks. *Chemical Geology* 160, 281–290.

Rose, E.F., Shimizu, N., Layne, G.D., Grove, T.L. (2001) Melt production beneath Mt. Shasta from boron data in primitive melt inclusions. *Science* 293, 281-283.

Rosner, M., Erzinger, J., Franz, G., Trumbull, R.B. (2003) Slab-derived boron isotope signatures in arc volcanic rocks from the Central Andes and evidence for boron isotope fractionation during progressive slab dehydration. *Geochemistry Geophysics Geosystems* 4, 9005.

Schiavi, F., Kobayashi, K., Nakamura, E., Tiepolo, M., Vannucci, R. (2011) Trace element and Pb–B–Li isotope systematics of olivine-hosted melt inclusions: insights into source metasomatism beneath Stromboli (southern Italy). *Contributions to Mineralogy and Petrology* 163, 1011–1031.

Shaw, A.M., Hauri, E.H., Behn, M.D., Hilton, D.R., Macpherson, C.G., Sinton, J.M. (2012) Long-term preservation of slab signatures in the mantle inferred from hydrogen isotopes. *Nature Geoscience* 5, 224-228.

Smith H.J., Spivack, A.J., Staudigel, H., Hart, S.R. (1995) The boron isotopic composition of altered oceanic crust. *Chemical Geology* 126, 119-135.

Smith, H.J., Leeman, W. P., Davidson, J., Spivack, A.J. (1997) The B isotopic composition of arc lavas from Martinique, Lesser Antilles. *Earth and Planetary Science Letters* 146, 303-314.

Spivack, A.J., Edmond, J.M. (1987) Boron isotope exchange between seawater and the oceanic crust. *Geochimica et Cosmochimica Acta* 51, 1033-1043.

Spivack, A.J., Palmer, M.R., Edmond, J.M. The sedimentary cycle of the boron isotopes. *Geochimica et Cosmochimica Acta* 51, 1939 (1987).

Spivack, A.J., You, C.-F., Smith, H.J. (1993) Foraminiferal boron isotope ratios as a proxy for surface ocean pH over the past 21 Myr. *Nature* 363, 149-151.

Straub, S.M., Layne, G.D. (2002) The systematics of boron isotopes in Izu arc front volcanic rocks. *Earth and Planetary Science Letters* 198, 25–39.

Tonarini, S., Leeman, W.P., Ferrara, G. (2001a) Boron isotopic variations in lavas of the Aeolian volcanic arc, South Italy. *Journal of Volcanology and Geothermal Research* 110, 155-170.

Tonarini, S., Armienti, P., D'Orazio, M., Innocenti, F. (2001b) Subduction-like fluids in the genesis of Mt. Etna magmas: evidence from boron isotopes and fluid mobile elements. *Earth and Planetary Science Letters* 192, 471-483.

Tonarini, S., Leeman, W.P., Civetta, L., D'Antonio, M., Ferrara, G., Necco, A. (2004) B/Nb and $\delta^{11}B$ systematics in the Phlegrean volcanic district. *Journal of Volcanology and Geothermal Research* 133, 123-139.

Tonarini, S., Agostini, S., Innocenti, F., Manetti P. (2005) $\delta^{11}B$ as a tracer of slab dehydration and mantle evolution in Western Analtolia Cenozoic magmatism. *Terra Nova* 17, 259-264.

Tonarini, S., Agostini, S., Doglioni, C., Innocenti, F., Manetti, P. (2007) Evidence for serpentinite fluid in convergent margin systems: the example of El Salvador (Central America) arc lavas. *Geochemistry, Geophysics, Geosystems* 8, Q09014.

Tonarini, S., D'Antonio, M., Di Vito, M.A., Orsi, G., Carandente, A. (2009) Geochemical and B-Sr-Nd isotopic evidence for mingling and mixing processes in the magmatic system that fed the Astroni volcano (4.1-3.8 ka) within the Campi Flegrei caldera (southern Italy). Lithos 107, 135-151.

Tonarini, S., Leeman, W.P., Leat, P.T. (2011) Subduction erosion of forearc mantle wedge implicated in the genesis ofo the South Sandwich Island (SSI) arc: evidence from boron isotope systematics. *Earth and Planetary Science Letters* 301, 275-284.

Turner, S., Tonarini, S., Bindeman, I., Leeman, W.P., Schaefer, B.F. (2007) Boron and oxygen isotope evidence for recycling of subducted components over the past 2.5 Gyr. *Nature* 447, 702-705.

Vengosh, A., Kolodny, Y., Starinsky, A., Chivas, A.R., McCulloch, M. T. (1991) Coprecipitation and isotopic fractionation of boron in modern biogenic carbonates. *Geochimica et Cosmochimica Acta* 55, 2901-2910.
